# The Common Follicle-Stimulating Hormone Receptor (FSHR) Promoter Polymorphism FSHR −29G > A Affects Androgen Production in Normal Human Small Antral Follicles

**DOI:** 10.3389/fendo.2017.00122

**Published:** 2017-06-02

**Authors:** Tanni Borgbo, Hana Klučková, Milan Macek, Jana Chrudimska, Stine Gry Kristensen, Lise Lotte Hansen, Claus Yding Andersen

**Affiliations:** ^1^Laboratory of Reproductive Biology, The Juliane Marie Centre for Women, Children and Reproduction, University Hospital of Copenhagen, Copenhagen, Denmark; ^2^Department of Biomedicine, University of Aarhus, Aarhus, Denmark; ^3^Department of Biology and Medical Genetics, 2nd Faculty of Medicine Charles University, University Hospital Motol, Prague, Czechia

**Keywords:** follicle fluid, follicle-stimulating hormone receptor −29G > A, follicle-stimulating hormone receptor polymorphisms, human small antral follicles, rs1394205

## Abstract

Follicle-stimulating hormone receptors (FSHRs) are almost exclusively expressed on granulosa cells, and FSH action is probably most clearly reflected in intrafollicular hormone milieu of antral follicles. Little is known about the possible effects of the common single nucleotide polymorphism (SNP) FSHR −29G > A (rs1394205) on hormonal conditions in humsan small antral follicles (hSAFs) obtained from women in the natural menstrual cycle. This study investigated the follicle fluid (FF) concentrations of anti-Müllerian hormone, estradiol, progesterone, androstenedione, and testosterone in hSAF in relation to the different genotypes of FSHR −29G > A. FF from 362 follicles was collected in 95 women undergoing fertility preservation, who did not suffer from a disease that directly affected ovarian function. The testosterone levels of the minor A/A genotype were significantly increased compared to the A/G and the G/G genotype. Furthermore, significantly reduced androstenedione levels were observed for the G/G genotype, as compared to the A/G genotype, while the other hormones did not show statistical significant differences. In conclusion, the androgen levels of hSAF were significantly elevated in the minor SNP genotype in the FSHR promoter polymorphism FSHR −29G > A.

## Introduction

FSH is instrumental in regulating ovarian activity and exerts a myriad of effects on follicles in all developmental stages through follicle-stimulating harmone receptors (FSHRs), which are almost exclusively expressed on granulosa cells (GCs). The number of FSHR proteins expressed on each GC change profoundly throughout follicular development. Low but measurable FSHR expression has been observed in human primordial follicles (1, 2), while maximal expression occurs in the antral stage declining toward ovulation (3). FSH signal transduction is effected *via* different pathways of which increase in intracellular cAMP production leading to activation of protein kinase A and transcription of a several downstream genes is one of the major ones (4–7). It is, however, currently unknown how FSHR density impacts on function and signal transduction.

Follicle-stimulating hormone receptors exist as a number of different genetic variants, i.e., polymorphisms, which may affect signal transduction. Three common single nucleotide polymorphisms (SNPs) have been identified in the FSHR gene: rs6165 (c.919A > G, p.Thr307Ala, FSHR 307), rs6166 (c.2039A > G, p.Asn680Ser, FSHR 680), and rs1394205 (c. −29G > A, FSHR −29G > A) (8–14).

The two most studied SNPs, FSHR 307, and FSHR 680, both reside on exon 10 in linkage disequilibrium. Studies have focused on the intrafollicular hormone milieu in human small antral follicles (hSAFs) (15) and especially their potential impact on ovarian stimulation (OS) have received a considerable interest (8–14, 16, 17). Although single studies has found an association to basic levels of FSH, the length of menstrual cycles, as well as the consumption of FSH used in OS and treatment outcome, no clear cut effects have been found (11, 14, 17–23). However, recently, Lindgreen and co-workers found a significant association to pregnancy rates especially in combination with a LH SNP (24).

The third common SNP FSHR −29G > A is located in the promoter region of FSHR and has been studied to a lesser extent. So far, studies have associated FSHR −29G > A to changes in FSHR gene expression levels, in which homozygote carriers of the minor allele (i.e., the A/A genotype) showed a reduced FSHR gene expression, compared to homozygote carriers of the major allele (i.e., the G/G genotype) (9, 10, 19). Furthermore, Desai and co-workers observed that carriers of the minor genotype required significantly higher amounts of exogenous FSH during OS, when compared to the G/G and G/A genotypes (9).

Potential differences in the signal transduction of the different FSHR polymorphisms is probably best studied in FF, as the concentrations of locally produced substances such as sex steroids and anti-Müllerian hormone (AMH) often shown concentrations up to a thousand times higher than in circulation (25–27). The aim of this study was to evaluate the impacts of the FSHR −29G > A genotype on the hormone profile in FF from hSAF.

## Materials and Methods

### Sample Material

In total, 362 follicles were collected from 95 women, with a mean age of 28.5 years (ranging from 15 to 38 years). The number of follicles analyzed from each woman ranged from 1 to 10 (mean 3.8 follicles/patient). Follicle diameters ranged from 3 to 13 mm, as calculated from the aspirated volume, assuming a spherical structure of the follicle. All follicles were obtained from surplus ovarian tissue from women undergoing fertility preservation, having ovarian tissue cryopreserved at Laboratory of Reproductive Biology, Rigshospitalet, Denmark. The diagnosis of women was in all cases unrelated to the ovary including polycystic ovary syndrome. The fertility preservation procedure involved excision of one entire ovary from which individual visible antral follicles were aspirated with a 23G needle attached to a syringe. The collection of FF had no effect on the fertility preservation procedure. In all cases, the ovary had overall gross normal appearance.

As the intrafollicular hormone milieu in human small antral follicles (hSAFs) are independent of circulating levels of gonadotropins and have no major impact on the serum hormone levels (15), serum hormone levels were not included in this study. The follicles were collected at various times during the menstrual cycle, as the dynamics of the hSAF are similar throughout the menstrual cycle (15, 28).

Parts of this material has been used in previous studies (3, 15, 27, 29, 30).

### Ethical Approval

The study was approved by the ethical committee of the municipalities of Copenhagen and Frederiksberg (H-2-2011-044). Oral information and written informed consent was obtained from research participants and from parents of minors.

### DNA Extraction

Seventy-two patients were genotyped using DNA extracted from approximately 25 mg ovarian tissue, using the DNeasy Blood & Tissue Kit (Qiagen, Hilden, Germany), following the manufacturers protocol. The DNA samples were subsequently stored at −20°C. The remaining 23 patients were genotyped from DNA extracted from aspirated GC, as no ovarian tissue was available for these patients. The DNA was extracted from the GC samples using Tri Reagent (Sigma-Aldrich, St. Louis, MO, USA), with slight modifications to the protocol as previously described (30).

### Genotyping of the FSHR −29G > A Polymorphism

The samples were genotyped using CADMA-based genotyping as previously published with slight modifications to the PCR mastermix (30). In brief, the final reaction mix consisted of 2 µl template DNA (10 ng/µl), 5 µl LightCycler^®^ 480 High Resolution Melting Master (Roche Diagnostics, Ropkreuz, Switzerland), 1.2 µl MgCl2 (25 µM, Roche Diagnostics, Ropkreuz, Switzerland), 0.2 µl wild-type primer 5′-TATGCATCCGTCCACCTGAGTTCTTC-3′ (10 µM), 0.2 µl mutation primer 5′-TATGCATCTATCCACATGATTTCTTT-3′ (10 µM), 0.2 µl common reverse primer 5′-GAGGTTTTTCTCTGCAAATGCAG-3′ (10 µM), and finally adding 1.2 µl ddH_2_O to a final volume of 10 µl.

### Hormone Measurements

The concentration of AMH, estradiol, progesterone, androstenedione, and testosterone were measured using commercially available ELISA assays. AMH was measured using the Ultra Sensitive AMH/MIS Elisa kit (Al-105-i, AnshLabs, Webster, TX, USA), according to the manufacturer’s protocol, with a 1:300 dilution of the FF samples using the supplied assay buffer. Estradiol, progesterone, androstenedione, and testosterone were initially measured using commercially available RIA kits (DSL-43100, DSL-3400, DSL-3800, DSL-4000; DSL, Webster, TX, USA). However, during the course of sample collection, the RIA assays became unavailable and it was necessary to switch to ELISA assays (NovaTec Immundiagnostica, Dietzenbach, Germany, DNOV 002, 003, 006 and 008, respectively).

Due to limited sample material, it was not possible to re-analyze the FF analyzed with RIA. The RIA kit was used to analyze between 11 and 15% of the FF samples in this study; progesterone (*n* = 39/336, 12%), estradiol (*n* = 37/337, 11%), testosterone (*n* = 47/338, 14%), and androstenedione (*n* = 50/345, 15%). Based on highly significant linear correlations between the results from the two types of assays (correlation coefficients: estradiol *r* = 0.99, progesterone *r* = 0.93, testosterone *r* = 0.91, and androstenedione *r* = 0.91), mathematical equations was used for transforming data from RIA into ELISA, as previously published (3). The remaining follicles were analyzed using NovaTech ELISA assays, according to the manufacturer’s protocol, using in-house prepared steroid-free serum for FF dilution.

### Statistical Analysis

The statistical analysis was performed using STATA version 13.1 (STATACorp LP, USA). The hormones were log- or square root-transformed in order to approximate normal distributions. A one-level mixed effects model was used for the statistical analysis, including follicle size as covariant and patients as random effect, in order to take into account that GC function varies with follicle size, and that follicles from one patient may be more similar than follicles from different patients. *P*-values ≤0.05 were considered statistically significant.

In this study, we have included follicles ranging from 3 to 13 mm in diameter. The intrafollicular hormone production is dependent on the developmental stage and size of the follicles, and a shift in hormone profiles is expected when follicles reach 810 mm when selection for dominance is initiated. In the follicle cohort of this study; however, no differences were found in the statistics, when excluding follicles above 8 mm (data not published).

## Results

The distribution between the three FSHR −29G > A genotypes (i.e., A/A, A/G, and G/G allele) was 14, 37, and 49%, respectively. This frequency distribution compares well to what have been reported from other countries (9, 13, 31).

**Figure 1:**
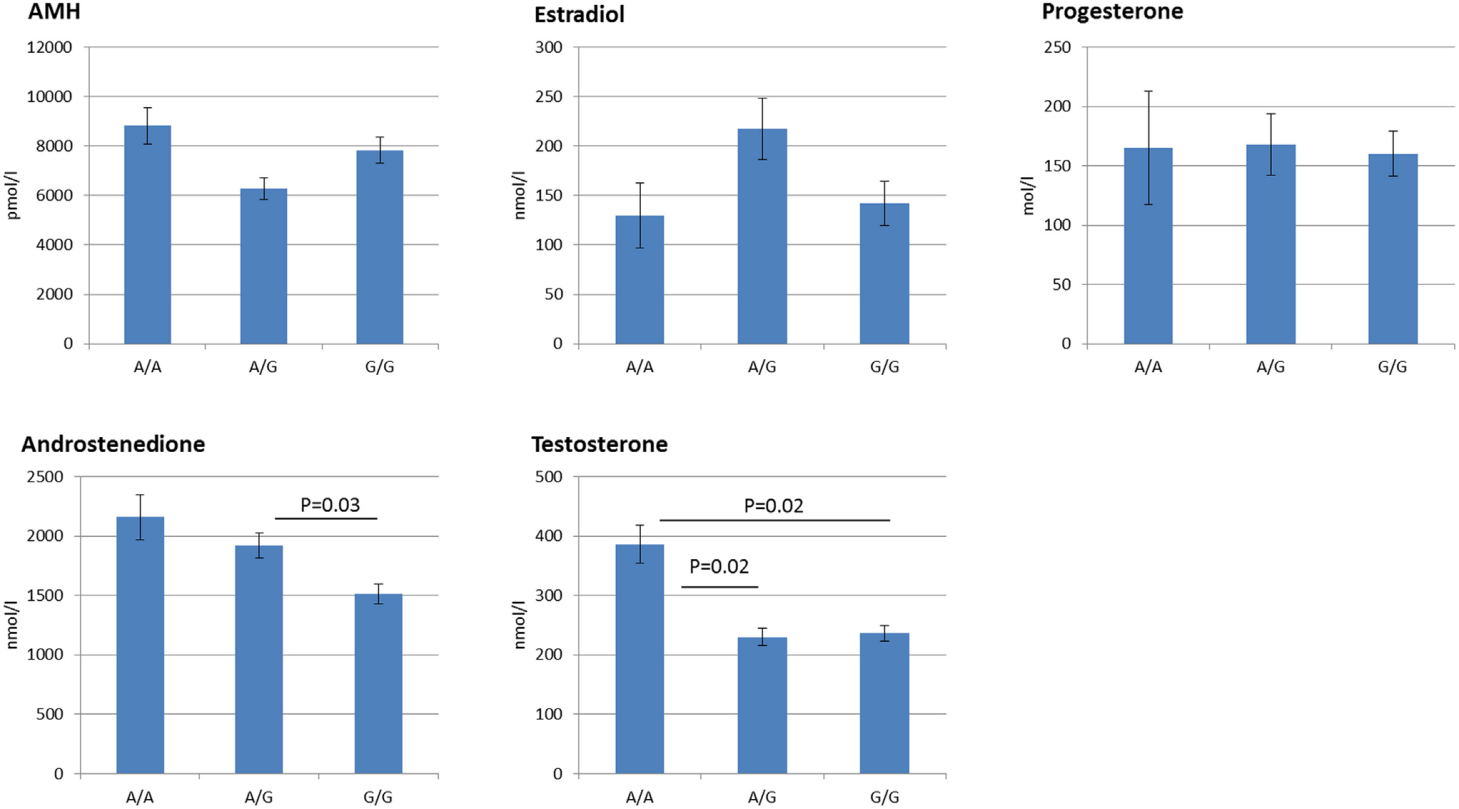
Follicular fluid hormone profiles grouped according to follicle stimulating hormone receptor −29G > A genotype. The figure displays the Mean ± SEM of the intrafollicular hormone levels. Statistically significant differences in hormone levels are observed for androstenedione, and testosterone. In addition, discrete genotype-dependent differences are observed for Anti-Müllerian hormone and estradiol in an inverse manner, although not statistically significant.

The FF hormone concentrations are summarized in Table 1 and Figure 1. Overall, the FSHR −29G > A genotype was found to be significantly associated to the androgen levels in hSAF. Significantly increased testosterone levels were observed for the A/A genotype as compared to both the A/G (*P* = 0.02) and G/G (*P* = 0.02) genotype. Likewise, significantly lower androstenedione levels were observed for the G/G genotype as compared to the A/G genotype (*P* = 0.03), however, with no significant differences observed between the A/A and the A/G genotype.

**Table 1:** Intrafollicular hormone levels grouped according to follicle-stimulating hormone receptor −29G > A genotype mean ± SEM.

Genotype	A/A			A/G			G/G		
	*n* follicle fluid (FF)	*n* (women)	Mean ± SEM	*n* (FF)	*n* (women)	Mean ± SEM	*n* (FF)	*n* (women)	Mean ± SEM
Anti-Müllerian hormone (pmol/l)	48	9	8,814 ± 746	133	37	6,266 ± 441	166	46	7,832 ± 510

Progesterone (mol/l)	49	10	165 ± 48	123	37	168 ± 26	164	46	160 ± 19

Estradiol (E_2_) (nmol/l)	48	10	130 ± 33	123	37	217 ± 31	166	47	142 ± 22

Testosterone (T) (nmol/l)	49	10	386 ± 32*^1,2^	125	37	230 ± 14*^1^	164	45	236 ± 13*^2^

Androstenedione (nmol/l)	49	10	2,157 ± 188	125	37	1,919 ± 104*^3^	171	47	1,521 ± 87*^3^

Age ±SD (years)		10	29.6 ± 5.8		37	28.8 ± 5.7		45	28.0 ± 5.9

Follicle diameter (mm)	50	10	5.3 ± 0.2	135	37	6.1 ± 0.1	177	48	5.6 ± 0.1

*Statistical significances.

*Testosterone: ^1^P = 0.02, ^2^P = 0.02*.

*Androstenedione: ^3^P = 0.03*.

No statistically significant differences were observed for AMH, estradiol, or progesterone.

## Discussion

On a relatively large sample size of more than 300 normal hSAF, the present study demonstrated that the minor genotype of the common SNP at position −29 of the FSHR promoter region, FSHR −29G > A, is significantly associated to augmented FF concentrations of androstenedione and testosterone. Androgens exert an important role in follicular development and in female reproduction, and the present results enforce that SNP’s in the gonadotropin receptors affect the fine-tuned pituitary gonadal interactions. The present study was performed entirely on hSAF from women who did not receive any kind of exogenous stimulation in their natural menstrual cycle and it remains to be seen whether the supraphysiological concentrations of FSH as used is connection with OS override these subtle differences and wipe out any impact on the reproductive output of fertility treatment.

Androgen production takes place exclusively in the theca cells, which do not express FSHR and there is no direct effect of FSH on theca cells. However, in rats, it has been shown that FSH-induced paracrine factors from GCs enhance theca cell androgen synthesis (32). Treatment with FSH was found to significantly increase the thecal CYP17A1 mRNA levels, as well as the androgen production in response to LH (32). Smyth and co-workers found that inhibin enhanced the LH-induced androgen production and suggested that FSH-induced paracrine factors produced by the GCs (e.g., inhibin-B or IGF2) could augment theca cell androgen output. More recently, we found a statistical significant association between inhibin-B and the androgen concentration in hSAF (33, 34). In the present study, inhibin-B was only measured in a small fraction of FF that did not allow a valid analysis.

Although the minor allele of FSHR −29G > A has been associated with reduced FSHR gene expression levels (9, 10, 19) and, therefore, would be expected to result in less paracrine androgen stimulation, it is not known how and if this specific SNP affect inhibin-B secretion. Especially, the inhibin α-gene has been shown to be upregulated by acidic FSH isoforms in contrast to almost any other FSH-inducible GC substance, where the less acidic isoforms are more potent (35). It could, therefore, also be speculated that the minor allele of FSHR −29G > A had a similar effect. It will, therefore, be of interest to evaluate the levels of inhibin-B and inhibin-A in FF from hSAF in order to evaluate whether levels of inhibin is also associated to this SNP.

The paracrine effects of androgens-enhancing GC FSHR expression has been documented in several studies (36, 37). Thus, GC from follicles obtained from women having the minor allele A/A genotype, which showed augmented androgen FF concentration, would be expected to show enhanced FSHR expression. The increased FSHR expression is expected to translate into an augmented aromatase expression. However, previous studies showed that the minor allele group had a reduced FSHR expression (9, 10, 19), which may explain why levels of estradiol are not significantly enhanced. It would be relevant to evaluate both the FSHR and LHR expression of the corresponding GCs to substantiate the effects of androgens in a future study.

In conclusion, this study shows that the minor A/A genotype at position −29 of FSHR is associated with an altered FF milieu, resulting in significant increased androgen levels.

## Ethics Statement

This study was carried out in accordance with the recommendations of the ethical committee of the municipalities of Copenhagen and Frederiksberg with written informed consent from all subjects. All subjects gave written informed consent in accordance with the Declaration of Helsinki. The protocol was approved by the ethical committee of the municipalities of Copenhagen and Frederiksberg (journal number; H-2-2011-044).

## Author Contributions

TB, CA, and MM designed the study and wrote the manuscript. TB performed the primer design for the CADMA-based FSHR assays, and performed the CADMA-based FSHR −29G > A genotyping. LH provided validating sequencing results of the CADMA-based FSHR assays. HK and JC performed validating FSHR −29G > A HRM genotyping. SK and CA collected follicular fluids, granulosa cells, and ovarian tissue for the study. TB performed the analysis and interpretation of the data, and prepared figures and tables for the manuscript. LH helped finalizing the manuscript.

## Conflict of Interest Statement

The authors declare that the research was conducted in the absence of any commercial or financial relationships that could be construed as a potential conflict of interest. The reviewer ST and handling Editor declared their shared affiliation, and the handling Editor states that the process nevertheless met the standards of a fair and objective review.
